# The efficacy and safety of combined GLP-1RA and basal insulin therapy among inadequately controlled T2D with premixed insulin therapy

**DOI:** 10.1097/MD.0000000000033167

**Published:** 2023-03-10

**Authors:** Jhih-Syuan Liu, Sheng-Chiang Su, Feng-Chih Kuo, Peng-Fei Li, Chia-Luen Huang, Li-Ju Ho, Kuan-Chan Chen, Yi-Chen Liu, Chih-Ping Lin, An-Che Cheng, Chien-Hsing Lee, Fu-Huang Lin, Yi-Jen Hung, Hsin-Ya Liu, Chieh-Hua Lu, Chang-Hsun Hsieh

**Affiliations:** a Division of Endocrinology and Metabolism, Department of Internal Medicine, Tri-Service General Hospital, National Defense Medical Center, Taipei, Taiwan, ROC; b School of Public Health, National Defense Medical Center, Taipei, Taiwan, ROC; c Institute of Preventive Medicine, National Defense Medical Center, Taipei, Taiwan, ROC; d BeYoung Research Institute.

**Keywords:** basal insulin, continuous glucose monitor system, glucagon-like peptide-1 receptor agonist, premixed insulin, type 2 diabetes mellitus

## Abstract

This study investigated the effect of a combination of glucagon-like peptide-1 receptor agonist (GLP-1 RA) and basal insulin (BI) in poorly controlled type 2 diabetes mellitus previously treated with premixed insulin. The possible therapeutic benefit of the subject is mainly hoped to provide a direction for optimizing treatment options to reduce the risk of hypoglycemia and weight gain. A single-arm, open-label study was conducted. The antidiabetic regimen was switched to GLP-1 RA plus BI to replace previous treatment with premixed insulin in type 2 diabetes mellitus subjects. After 3 months of treatment modification, GLP-1 RA plus BI was compared for superior outcomes by continuous glucose monitoring system. There were 34 subjects at the beginning, 4 withdrew due to gastrointestinal discomfort, and finally 30 subjects completed the trial, of which 43% were male; the average age was 58 ± 9 years old, and the average duration of diabetes was 12 ± 6 years, the baseline glycated hemoglobin level was 8.6 ± 0.9 %. The initial insulin dose of premixed insulin was 61 ± 18 units, and the final insulin dose of GLP-1 RA + BI was 32 ± 12 units (*P* < .001). Time out of range (from 59%–42%), time-in-range (from 39%–56%) as well as glucose variability index including standard deviation also improved, mean magnitude of glycemic excursions, mean daily difference and continuous population in continuous glucose monitoring system, continuous overall net glycemic action (CONGA). Also noted was a decrease in body weight (from 70.9 kg–68.6 kg) and body mass index (all *P* values < .05). It provided important information for physicians to decide to modify therapeutic strategy as individualized needs.

## 1. Introduction

The incidence and prevalence of type 2 diabetes mellitus (T2D) has been growing worldwide recently.^[1]^ Owing to progressive nature of T2D, especially decline of insulin secretion,^[2]^ many patients eventually require insulin therapy usually initialized with a long-acting (basal) formulation daily according to current guideline.^[3]^ There are 3 therapeutic strategies of intensified injectable therapy when failed in oral antidiabetic agents and basal insulin therapy, which including adding short-acting insulin analogue (basal plus or basal-bolus), or adding GLP 1RA, or shifting to premixed insulin with individualized advantages and shortages in current treatment guideline.^[4]^ The choice of treatment regimen may base on efficacy, side effects of drugs (including hypoglycemia and weight gain), patient’s preference, and atherosclerotic cardiovascular risk benefits. Furthermore, glucose variability (GV) is a glucose index via continuous glucose monitoring system (CGMS) and closely correlated to diabetic complications according to the previous studies.^[5,6]^

If basal insulin has been titrated to an acceptable fasting blood glucose without optimal glycated hemoglobin (HbA1C) level, treatment may be intensified by changing to premixed insulin formulations. Moreover, flexible premixed insulin therapy regimens were the better option for these patients to cover postprandial insulin need.^[7]^ However, clinical inertia among clinical physicians and patients, would be difficult to control hyperglycemia with complex therapeutic formula and/or lack of patient compliance. Therefore, premixed insulins contain fixed percentages of intermediate and short acting, may cause hardly dosage adjustment. It has also led not only to more events of hyperglycemia and hypoglycemia rebound hyperglycemia (Somogyi effect),^[8]^ but also higher GV.^[9]^ Body weight gain is also another expected result for long-tern use of insulin.^[10]^ Glucagon-like peptide-1 receptor agonists (GLP-1 RA), 1 hormone of incretin families, can enhance glucose-dependent insulin secretion and suppress glucagon production of pancreas.^[11]^ GLP-1 RAs are also attractive options for the treatment of T2D because they effectively lower HbA1C fasting plasma glucose, postprandial glucose, and body weight.^[12]^ Therefore, GLP-1 RAs have a low risk of hypoglycemia and stable GV.^[13]^ Not like insulin, GLP-1 RAs have fixed dosage injection to control sugar and rarely monitor blood sugar.^[14]^ The combination of GLP-1 RA plus basal insulin has been proved as a treatment option to intensify insulin therapy in T2D. The benefits of combined therapy include lowering serum glucose and body weight, and comfortable accessibility.^[15,16]^ However, it is unclear whether T2D patients who have previously received premixed insulin therapy and poor blood sugar control can increase the compliance of injection therapy due to the enhanced control of blood sugar and body weight through the combined injection of GLP-1-RA and basal insulin.

The purpose of this study was to evaluate the feasibility, efficacy, safety, and GV of using combined injectable GLP1-RA and basal insulin therapy instead of twice-daily premixed insulin therapy in poorly controlled T2D patients.

## 2. Materials and methods

### 2.1. Subjects and study design

The subjects were enrolled form the outpatient department of Tri-Service General Hospital between January 2018 and October 2019. All study subjects were anonymous, and informed consent was obtained prior to participation. The study proposal was reviewed and approved by the institutional review board of Tri-Service General Hospital

It is a single-arm, open, observational study. The subjects with documented T2D diagnosis longer than 3 months with aged from 40 to 80 with HbA1C levels between 7.0% to 11.0% under treatment of premixed insulin, NovoMix® 30 (30% insulin aspart and 70% insulin aspart protamine) with or without combination with metformin were enrolled into the study. The patients with Alanine transaminase (ALT) and Aspartate transaminase (AST) > 3 times normal, and estimated GFR < 30mL/minute/1.73m^2^, or major systemic disease were excluded from the study.

After enrollment, patients were scheduled for laboratory tests and insertion of CGMS after an 8 to 10 hours NPO. Patients were kept treating with premixed insulin for another week during CGMS insertion by experienced staff. After 1 week, antidiabetic regimen was changed to insulin glargine with an initial dose 40% to 50% of the previous total daily dose of premixed insulin. At the same time liraglutide was also started with an initial dose of 0.6 mg/day with subsequent up-titration to 1.2 mg/day after 1 week, if well tolerated. Repaglinide 1 to 2 mg 3 times per day were prescribed to reach the goal of postprandial glucose level < 180 mg/dL. Insulin glargine dose was regularly up-titrated at weekly interval according to fasting plasma glucose to reach goal of 90 to 130 mg/dL or reaching insulin dose of 50% of patient’s weight. After a total treatment duration of 12 weeks, another CGMS procedure were performed again The glycemic index, clinical cardiovascular risk profiles, safety issues (body weight and hypoglycemia), and GV indices from CGMS before and after 3 months treatment modification was evaluated.

Body mass index (BMI) was calculated as body weight (kg)/height (m2). Systolic blood pressure and diastolic blood pressure were measured in the right arm of seated individuals by using a standard mercury sphygmomanometer.

### 2.2. Laboratory measurements

After maintaining a fasting state for 12 hours, blood samples were obtained from each participant to measure plasma glucose, HbA1C, creatinine, and lipid profiles. Serum total cholesterol, triglyceride, and low-density lipoprotein cholesterol (LDL-C) were determined using the dry, multilayer analytical slide method in the Fuji Dri-Chem 3000 analyzer (Fuji Photo Film Corporation, Tokyo, Japan). The levels of HbA1C were evaluated by ion-exchange high-pressure liquid chromatography method (BIO-RAD VARIANT II, Los Angeles, CA). Plasma glucose concentrations were determined by the glucose oxidase method on a Beckman Glucose Analyzer II (Beckman Instruments, Fullerton, CA).

### 2.3. Details of CGM insertion (iPRO 2™ CGM system, MMT-7741, medtronic) and glucose variability indices

CGM measures glucose in interstitial fluid through subcutaneous sensor and saves data in the recorder every 5 minutes. It has been validated by several studies and was also known to provide a very well correlation between blood and interstitial fluid glucose values.^[17]^ A minimum of 3 self-monitoring of blood glucose values per day from glucometer are needed to calibrate the glucose sensor data. CGM measurements provided several important information of GV, including time per day within target glucose range (time-in-range, glucose levels between 70–180 mg/dL), time-above target glucose range (time-above-range, glucose levels > 180 mg/dL), and time-below target glucose range (Time-Below-Range, glucose levels < 70 mg/dL). GV indices, which included standard deviation (SD), (magnitude of glycemic excursions [MAGE], average of blood glucose excursions exceeding 1 SD of the mean blood glucose value), (mean of daily differences [MODD], the absolute difference between the paired CGMS values obtained during 2 successive days (minimum and maximum SD days), and (continuous overall net glycemic action [CONGA], SD of differences between observed blood glucose reading and an observed blood glucose level).^[18,19]^

### 2.4. Statistical analysis

Arithmetic means and standard deviations (SD) were calculated for the variables measured at least on an interval scale. Categorical data were presented as numbers (n) and percentages (%). For changes in clinical characteristics, biochemistry profiles, and CGM data at 2 different study points, Paired *t* test or Wilcoxon-Signed rank test was performed as applicable. Spearman correlation coefficients, with the changes of CGM record as dependent variables, was used to study the association and independent determinants of covariates. A *P* value of < 0.05 was considered to be statistically significant. All statistical analyses were performed using SPSS Inc. 26.0 software (SPSS, Chicago, IL). The number of patients reporting adverse events during the different treatments was recorded.

## 3. Results

**Figure 1.**
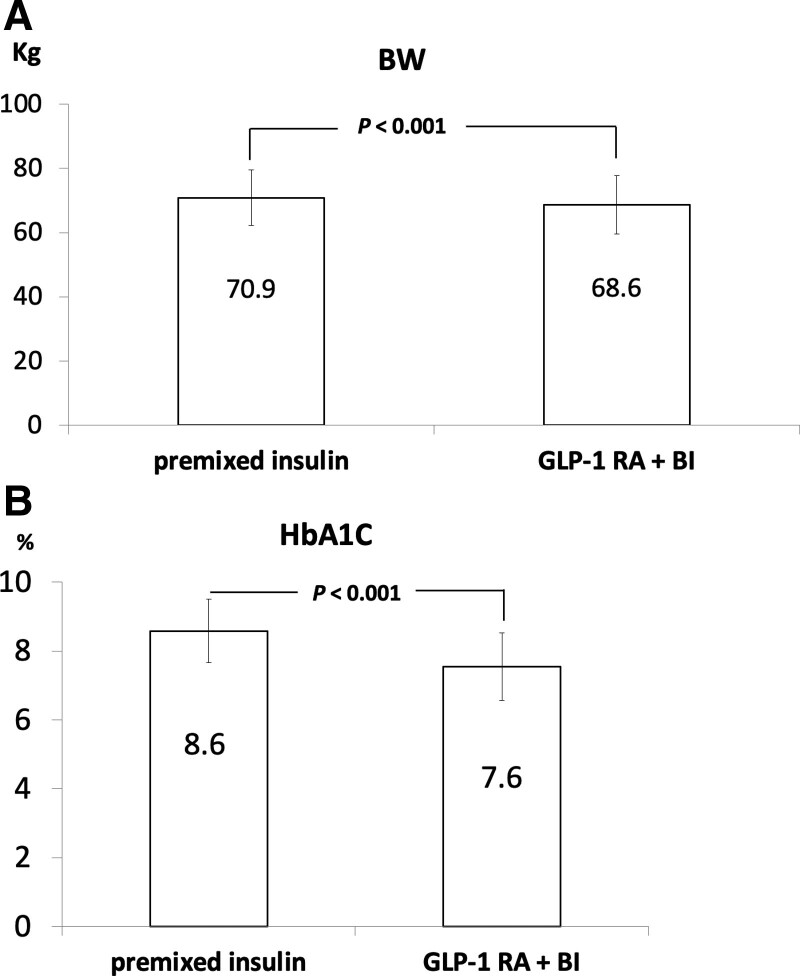
Changes of BW (1A) and HbA1C (1B) in premixed insulin and GLP-1 RA + BI. BI = basal insulin, GLP-1 RA = glucagon-like peptide-1 receptor agonist, HbA1c = glycated hemoglobin.

**Figure 2.**
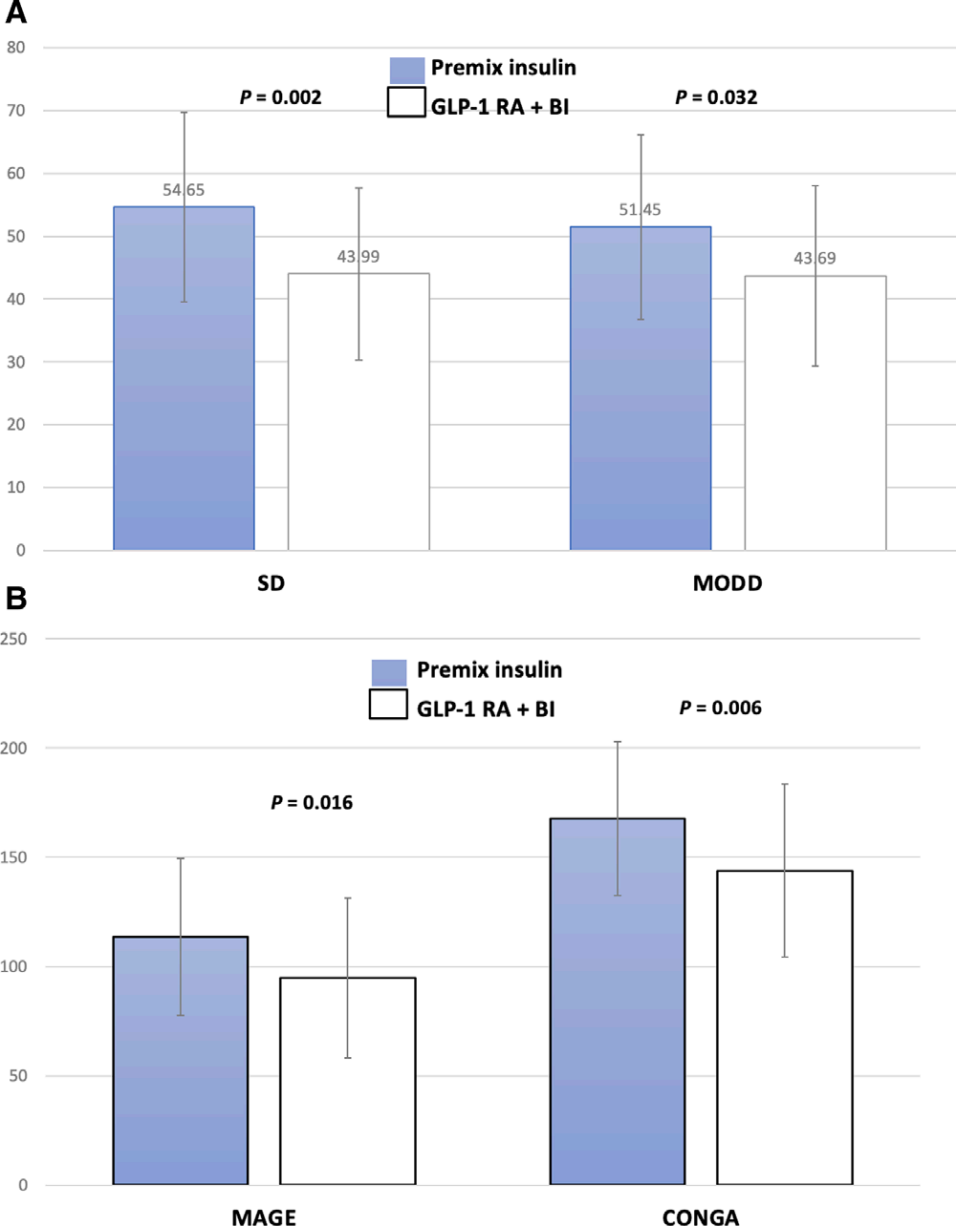
Changes of standard deviation (SD) (2A), mean of daily differences (MODD) (2A); mean amplitude of glycemic excursions (MAGE) (2B), and continuous overall net glycemic action (CONGA) (2B) in premixed insulin and GLP-1 RA + BI. BI = basal insulin, CONGA = continuous overall net glycemic action, GLP-1 RA = glucagon-like peptide-1 receptor agonist, MAGE = mean amplitude of glycemic excursions, MODD = mean of daily differences, SD = standard deviation.

As shown in Table 1, we enrolled a total of 34 patients with T2D who received premix insulin and changed it to GLP-1RA + basal insulin. Four of them quit the trial project because of gastrointestinal discomfort caused by GLP-1RA. Finally, a total of 30 subjects with 43% males; mean age of 58 ± 9 years, mean diabetes duration of 12 ± 6 years and baseline HbA1C level of 8.6 ± 0.9 % were included. There were a total of 14 people with an average dose of 1321 mg daily, whether to change the needle or continue to use metformin during the treatment, and the dose of metformin remained unchanged. After modification of treatment strategy, there were statistical significances in reduction of body weight 2.0 kg (*P* < .001), HbA1C of 1.0% (*P* < .001) (Fig. 1A and B), and BMI of 0.9 kg/m^2^ (*P* < .001), lowing fasting plasma glucose of 35 mg/dL (*P* = .004), and LDL-C of 11 mg/dL (*P* = .012) (Table 1). The initial dose of premixed insulin was 61 ± 18 units, and the final dose of GLP-1 RA + basal insulin was 32 ± 12 units. There was a statistically significant difference in the final dosage of insulin between the 2 groups, with a *P* value of < .001(Table 1). There were improved estimated HbA1c by 1% (*P* = .018), time-above-range by 17% (*P* = .002), time-in-range by 17% (*P* = .001) (Table 1). In Figure 2A and B, the data showed the end results of the study that improved of standard deviation (SD) of 11 (*P* = .002), MODD by 7 mg/dL (*P* = .032), MAGE by 18 mg/dL (*P* = .016), and CONGA of 19.8 mg/dL (*P* = .006). However, time-below-range (TBR) was not different between these 2 treatment strategies. Moreover, the improvement of HbA1C level is significantly positive correlation to narrowing range of main index about GV (SD, MAGE, MODD, and CONGA) (*R* = 0.481, 0.495, 0.584, and 0.623) (*P* = .007, 0.005, 0.001, and < 0.001) (Table 2).

**Table 1:** Baseline characteristics, biochemistry profiles, and CGMS data of study subjects with premixed insulin (before) and GLP-1 RA with basal insulin (after).

All	premixed insulin	GLP-1 RA + BI	*P* value
	(n = 30)	(n = 30)	
Males (n,%)	13(43)	-	-
Age (yr-old)	58 ± 10	-	-
Diabetes duration (yr)	13 ± 7	-	-
Statin use (%)	83	-	-
Metformin use (%)	47	-	-
& dose (mg)	1321	-	-
TDD of insulin (IU)	61 ± 18	32 ± 12	<.001
BMI (Kg/m^2^)	27.7 ± 3.2	26.8 ± 3.3	<.0001
SBP (mm Hg)	136 ± 16	132 ± 16	.086
DBP (mm Hg)	78 ± 9	78 ± 9	.907
FPG (mg/dL)	174 ± 68	139 ± 41	.004
TC (mg/dL)	151 ± 82	141 ± 75	<.001
LDL-C (mg/dL)	84 ± 18	76 ± 21	.021
TG (mg/dL)	147 ± 79	139 ± 71	.3291
Cr (mg/dL)	0.93 ± 0.38	0.92 ± 0.41	.541
eGFR (mL/min/1.73m^2^)	87 ± 30	89 ± 30	.258
Estimated HbA1C (%)	8.0 ± 1.3	7.0 ± 1.5	.018
TAR (%)	59 ± 19	42 ± 29	.002
TIR (%)	39 ± 18	56 ± 28	.001
TBR (%)	1.9 ± 3.5	1.9 ± 3.2	.947

BI = basal insulin, BMI = body mass index, CGMS = continuous glucose monitoring system, Cr = creatinine, DBP = diastolic blood pressure, eGFR = estimated glomerular filtration rate, FPG = fasting plasma glucose, GLP-1 RA = glucagon-like peptide-1 receptor agonist, HbA1c = glycated hemoglobin, LDL − C = low-density lipoprotein − cholesterol, SBP = systolic blood pressure, TAR = time-above-range, TBR = time-below-range, TC = total cholesterol, TDD = total daily dose, TG = Triglyceride, TIR = time-in-range.

**Table 2:** Spearman correlation coefficients between the change of HbA1c and the difference of variables in CGMS on premixed insulin (before) and GLP-1 RA + BI (after) treatment among study subjects.

ΔHbA1c	*r*	*P* value
ΔSD	0.481	.007
ΔMAGE	0.495	.005
ΔMODD	0.584	.001
ΔCONGA	0.623	<.001

BI = basal insulin, CGMS = continuous glucose monitoring system, CONGA = continuous overall net glycemic action, GLP-1 RA = glucagon-like peptide-1 receptor agonist, HbA1c = glycated hemoglobin, MAGE = mean amplitude of glycemic excursions, MODD = mean of daily differences, SD = standard deviation.

## 4. Discussion

In this observational study, we found that combined basal insulin plus GLP1 RA therapy achieved HbA1C level improvement and weight reductions, diminished glucose fluctuation with safety profiles than premixed insulin regimen among patients of T2D who needed intensification to injectable therapy. It provided clinical judgement for physician when treating patients with T2D individually.

The progressive nature course of T2D indicates that many individuals need multiple therapeutic strategies to maintain optimal glycemic targets.^[20,21]^ Updated guidelines from the American Diabetes Association and American Association of Clinical Endocrinologists recommend to consider a combination of GLP-1 RA therapies in T2D with high risks or established atherosclerotic cardiovascular disease, independent on individualized HbA1C target.^[4,22]^ Moreover, basal insulin is the most convenient initial insulin regimen and can be add-on to metformin and other antidiabetic agents.^[23]^ The combination injectable therapy of GLP-1 RA and basal insulin gets powerful glucose-lowering function and minimize weight gain and hypoglycemic events, comparing with premixed insulin twice-daily regimen.^[18,24]^

Our study concept is also similar to recently published studies, except that they used iGlaLixi fixed combination therapy.^[25,26]^ Similar to our results, body weight, HbA1c, and therapeutic total insulin dose can be adjusted by changing the therapeutic strategy improved by GLP-1RA plus basal insulin; and also did not increase the risk of hypoglycemia.

Our results combined GLP1RA and basal insulin showed beneficial lipid profiles than premixed insulin in T2D subjects. This benefit comes from therapy with GLP1RA. GLP-1 can also regulate cholesterol and triglycerides by several different pathways. GLP-1R signaling could decrease VLDL-TG production from liver, reduces TG content by modulating key hepatic enzymes of lipid metabolism, and interferes hepatocyte de novo lipogenesis and lipid β-oxidation, as well as, also could modify reverse cholesterol transport.^[27]^ Another study revealed that the BMI was significantly reduced by the GLP-1 RA treatment, however, the degree of LDL-C reduction was not correlation with that of BMI in the GLP-1RA subjects regardless of statin use.^[28]^ In our study, we reported significant reductions in total and LDL-C for previous premixed insulin treated T2D after shifting to a combination therapy of GLP1-RA and basal insulin. This mechanism for the lowering level of LDL-C is still exactly unclear, but might be partially related to weight loss and less oral-intake. Furthermore, some factors other than GLP-1 effect such as intensive lifestyle factors may influence the result.

GV is an alternative marker for chronic diabetic complications for patients of T2D. In the VARIATION study, T2D patients under combination therapy of basal insulin with a GLP-1 RA reach well glycemic control and the lowest glucose variability, comparing with subjects with other common insulin therapy regimens including premixed contents. Furthermore, TBR of CGM among combined treatment group is significantly lower than a premixed insulin treated 1.^[29]^ It similar in our research, where indicators (SD, MAGE, MODD, CONGA) of GV in CGM as well as biochemical and estimated HbA1C level were improved. The complementary effects of the combination of GLP-1 RA with basal insulin, lowering both postprandial and fasting hyperglycemia,^[30]^ may lead to narrowing GV for this combination strategy. Nevertheless, in our research, TBR of CGM between premixed insulin and combined treatment groups did not significantly change (Table 1). We find that our study subjects had higher HbA1C level (HbA1C 8.6% in our study vs 6.9%–7.0% in VARIATION trial) under premixed insulin control with insufficient dosage adjustment. It might be explained that they had a relatively higher sugar level all the time and then led to fewer hypoglycemic events. However, combination therapy of basal insulin and GLP-1 RA still had low episodes of hypoglycemia in both studies (1.9 %).

High GV means wide fluctuation in glucose levels result in acute change among hyperglycemia and hypoglycemia, may cause significant clinical complications.^[31,32]^ Several studies demonstrated that diet control, exercise, and antidiabetic medication could stabilize GV and minimize of hypoglycemia, resulting in improving a series of vascular injury responses.^[33,34]^ The FLAT-SUGAR Trial also concluded that GLP-1 RA with basal insulin minimized GV, reduced body weight, and decreased several cardiometabolic risk markers, comparing with basal-bolus insulin regimen therapy, without correlation to HbA1C change.^[15]^ However, there was still limited data to explore the correlation between improvement of HbA1C and GV previously. In clinical, they’re important markers for evaluation of glycemic control of DM, but independent means for each other. In our study, it an interesting finding, but difficult to be explained. We hypothesize that pleiotropic effect of GLP-1 could reach both target of optimal level of HbA1C and range of GV.^[35,36]^ In addition, basal insulin component with Neutral Protamine Hagedorn (NPH) shifting to insulin glargine U300 could also share the same benefits.^[37,38]^ However, further studies for the phenomenon should be surveyed in the future.

Several limitations in our study were noted. The observational study may be considered first, we only record separately once HbA1C level and CGM data in groups under premixed insulin (before) and GLP-1 RA + basal insulin (after) treatment within 3 months, thus the results should be interpreted with caution. Second, enrolled patients had a different degree of diabetes education across the study. For example, few study subjects not always adjusted the basal and premixed insulin dose throughout the study to achieve optimal pre- or postprandial sugar levels. Next, we did not indicate unique 1 research staff, blinded to study assignment and projects, to download the CGM data, when a separate research associate was responsible for participants’ evaluation and data collection. And then, another limitation is that we could not assure the patients adherence to diet control, exercise, and medication although we prove that they did regular visit during the OPD follow-up. After that, the results of this study were obtained in an only-Chinese population in Taiwan. Thus, the generalization of our result to other populations is limited. Finally, a weak point of this study is the smaller number of subjects included, which makes generalized results not really confident. In the future, large study analyses are necessary to account for the combined GLP-1 RA and basal insulin therapy comparing with multiple dose injection of premixed insulin treatment. Nevertheless, our study was only designed to confirm the principle of safe and therapeutic potential of combined GLP1-RA and long-acting basal insulin, which was illustrated in our results.

In summary, combined both GLP-1 RA and basal insulin therapy showed a significant improvement of glycemic indices, cardiovascular benefits, and GV among patients with uncontrolled T2D on previous therapy with premixed insulin. It provided important information for physicians to choose suitable therapeutic strategy as individualized needs. A larger scaled study is necessary to be conducted to validate these findings.

## Acknowledgments

The authors thank all individuals who participated in the study.

## Author contributions

**Conceptualization:** Sheng-Chiang Su, Feng-Chih Kuo, Peng-Fei Li, Chia-Luen Huang, Li-Ju Ho, Kuan-Chan Chen, Yi-Chen Liu, Chih-Ping Lin, An-Che Cheng, Chien-Hsing Lee, Yi-Jen Hung, Hsin-Ya Liu.

**Formal analysis:** Fu-Huang Lin.

**Writing – original draft:** Jhih-Syuan Liu.

**Writing – review & editing:** Chieh-Hua Lu, Chang-Hsun Hsieh.

## References

[1] Cho NH Shaw JE Karuranga S. IDF diabetes atlas: global estimates of diabetes prevalence for 2017 and projections for 2045. Diabetes Res Clin Pract. 2018;138:271–81. 29496507 10.1016/j.diabres.2018.02.023

[2] U.K. prospective diabetes study 16. Overview of 6 years’ therapy of type II diabetes: a progressive disease. U.K. prospective diabetes study group. Diabetes. 1995;44:1249–58. 7589820

[3] Davies MJ D’Alessio DA Fradkin J. Management of hyperglycemia in type 2 diabetes, 2018. A consensus report by the American Diabetes Association (ADA) and the European Association for the Study of Diabetes (EASD). Diabetes Care. 2018;41:2669–701. 30291106 10.2337/dci18-0033 PMC6245208

[4] Draznin B Aroda VR. 9. Pharmacologic approaches to glycemic treatment: standards of medical care in diabetes-2022. Diabetes Care. 2022;45:S125–S43. 34964831 10.2337/dc22-S009

[5] Ilag LL Kerr L Malone JK. Prandial premixed insulin analogue regimens versus basal insulin analogue regimens in the management of type 2 diabetes: an evidence-based comparison. Clin Ther. 2007;29 Spec No:1254–70. 18046926

[6] Rosenstock J Ahmann AJ Colon G. Advancing insulin therapy in type 2 diabetes previously treated with glargine plus oral agents: prandial premixed (insulin lispro protamine suspension/lispro) versus basal/bolus (glargine/lispro) therapy. Diabetes Care. 2008;31:20–5. 17934150 10.2337/dc07-1122

[7] Vaag A Lund SS. Insulin initiation in patients with type 2 diabetes mellitus: treatment guidelines, clinical evidence and patterns of use of basal versus premixed insulin analogues. Eur J Endocrinol. 2012;166:159–70. 21930715 10.1530/EJE-11-0022 PMC3260696

[8] Raskin P Allen E Hollander P. Initiating insulin therapy in type 2 diabetes: a comparison of biphasic and basal insulin analogs. Diabetes Care. 2005;28:260–5. 15677776 10.2337/diacare.28.2.260

[9] Turner HE Matthews DR. The use of fixed-mixture insulins in clinical practice. Eur J Clin Pharmacol. 2000;56:19–25. 10853873 10.1007/s002280050715

[10] Raccah D Bretzel RG Owens D. When basal insulin therapy in type 2 diabetes mellitus is not enough--what next? Diabetes Metab Res Rev. 2007;23:257–64. 17315242 10.1002/dmrr.733

[11] Testa MA Gill J Su M. Comparative effectiveness of basal-bolus versus premix analog insulin on glycemic variability and patient-centered outcomes during insulin intensification in type 1 and type 2 diabetes: a randomized, controlled, crossover trial. J Clin Endocrinol Metab. 2012;97:3504–14. 22851487 10.1210/jc.2012-1763

[12] Huang Y Heng C Wei J. Influencing factors of glycemic variability in hospitalized type 2 diabetes patients with insulin therapy: a Strobe-compliant article. Medicine (Baltim). 2017;96:e8021. 10.1097/MD.0000000000008021 PMC6392839 28885369

[13] Campbell JE Drucker DJ. Pharmacology, physiology, and mechanisms of incretin hormone action. Cell Metab. 2013;17:819–37. 23684623 10.1016/j.cmet.2013.04.008

[14] Garber AJ. Long-acting glucagon-like peptide 1 receptor agonists: a review of their efficacy and tolerability. Diabetes Care. 2011;34:S279–84. 21525469 10.2337/dc11-s231 PMC3632193

[15] O’Brien KD Hirsch IB Riddle MC. Response to comment on the FLAT-SUGAR trial investigators. glucose variability in a 26-week randomized comparison of mealtime treatment with rapid-acting insulin versus GLP-1 agonist in participants with type 2 diabetes at high cardiovascular risk. Diabetes Care. 2016;39:e188. 27208320 10.2337/dc15-2782

[16] Cho YM Wideman RD Kieffer TJ. Clinical application of glucagon-like Peptide 1 receptor agonists for the treatment of type 2 diabetes mellitus. Endocrinol Metab (Seoul). 2013;28:262–74. 24396690 10.3803/EnM.2013.28.4.262 PMC3871042

[17] Berlie H Hurren KM Pinelli NR. Glucagon-like peptide-1 receptor agonists as add-on therapy to basal insulin in patients with type 2 diabetes: a systematic review. Diabetes Metab Syndr Obes. 2012;5:165–74. 22826635 10.2147/DMSO.S27528 PMC3402010

[18] Eng C Kramer CK Zinman B. Glucagon-like peptide-1 receptor agonist and basal insulin combination treatment for the management of type 2 diabetes: a systematic review and meta-analysis. Lancet. 2014;384:2228–34. 25220191 10.1016/S0140-6736(14)61335-0

[19] Siegelaar SE Holleman F Hoekstra JB. Glucose variability; does it matter? Endocr Rev. 2010;31:171–82. 19966012 10.1210/er.2009-0021

[20] Defronzo RA. Banting Lecture. From the triumvirate to the ominous octet: a new paradigm for the treatment of type 2 diabetes mellitus. Diabetes. 2009;58:773–95. 19336687 10.2337/db09-9028 PMC2661582

[21] Schernthaner G Barnett AH Betteridge DJ. Is the ADA/EASD algorithm for the management of type 2 diabetes (January 2009) based on evidence or opinion? A critical analysis. Diabetologia. 2010;53:1258–69. 20352408 10.1007/s00125-010-1702-3 PMC2877312

[22] Garber AJ Handelsman Y Grunberger G. Consensus statement by the American association of clinical endocrinologists and American college of endocrinology on the comprehensive type 2 diabetes management algorithm - 2020 executive summary. Endocr Pract. 2020;26:107–39. 32022600 10.4158/CS-2019-0472

[23] Barnett AH. A review of basal insulins. Diabet Med. 2003;20:873–85. 14632712 10.1046/j.1464-5491.2003.00996.x

[24] Maiorino MI Chiodini P Bellastella G. Insulin and glucagon-like peptide 1 receptor agonist combination therapy in type 2 diabetes: a systematic review and meta-analysis of randomized controlled trials. Diabetes Care. 2017;40:614–24. 28325801 10.2337/dc16-1957

[25] Chen X Xu Y Zhang J. Exenatide twice daily plus glargine versus Aspart 70/30 twice daily in patients with type 2 diabetes with inadequate glycemic control on premixed human insulin and metformin. Endocr Pract. 2021;27:790–7. 33831552 10.1016/j.eprac.2021.03.015

[26] Gomez-Peralta F Al-Ozairi E Jude EB. Titratable fixed-ratio combination of basal insulin plus a glucagon-like peptide-1 receptor agonist: a novel, simplified alternative to premix insulin for type 2 diabetes. Diabetes Obes Metab. 2021;23:1445–52. 33651460 10.1111/dom.14365 PMC8252507

[27] Patel VJ Joharapurkar AA Shah GB. Effect of GLP-1 based therapies on diabetic dyslipidemia. Curr Diabetes Rev. 2014;10:238–50. 24998439 10.2174/1573399810666140707092506

[28] Hasegawa Y Hori M Nakagami T. Glucagon-like peptide-1 receptor agonists reduced the low-density lipoprotein cholesterol in Japanese patients with type 2 diabetes mellitus treated with statins. J Clin Lipidol. 2018;12:62–69.e1. 29217412 10.1016/j.jacl.2017.11.006

[29] Bajaj HS Venn K Ye C. Lowest glucose variability and hypoglycemia are observed with the combination of a GLP-1 receptor agonist and basal insulin (VARIATION Study). Diabetes Care. 2017;40:194–200. 27913575 10.2337/dc16-1582

[30] Anderson SL Trujillo JM. Basal insulin use with GLP-1 receptor agonists. Diabetes Spectr. 2016;29:152–60. 27574369 10.2337/diaspect.29.3.152 PMC5001217

[31] Smith-Palmer J Brandle M Trevisan R. Assessment of the association between glycemic variability and diabetes-related complications in type 1 and type 2 diabetes. Diabetes Res Clin Pract. 2014;105:273–84. 25023992 10.1016/j.diabres.2014.06.007

[32] Skrha J Soupal J Skrha J Jr.. Glucose variability, HbA1c and microvascular complications. Rev Endocr Metab Disord. 2016;17:103–10. 26975588 10.1007/s11154-016-9347-2

[33] Figueira FR Umpierre D Casali KR. Aerobic and combined exercise sessions reduce glucose variability in type 2 diabetes: crossover randomized trial. PLoS One. 2013;8:e57733. 23536769 10.1371/journal.pone.0057733 PMC3594238

[34] Colmegna PH Sanchez-Pena RS Gondhalekar R. Reducing glucose variability due to meals and postprandial exercise in T1DM using switched LPV control: in silico studies. J Diabetes Sci Technol. 2016;10:744–53. 27022097 10.1177/1932296816638857 PMC5038547

[35] Mori Y Taniguchi Y Sezaki K. Liraglutide narrows the range of circadian glycemic variations in Japanese type 2 diabetes patients and nearly flattens these variations in drug-naive type 2 diabetes patients: a continuous glucose monitoring-based study. Diabetes Technol Ther. 2011;13:1139–44. 21877924 10.1089/dia.2011.0137

[36] Matsumoto S Yamazaki M Kadono M. Effects of liraglutide on postprandial insulin and glucagon responses in Japanese patients with type 2 diabetes. J Clin Biochem Nutr. 2013;53:68–72. 23874074 10.3164/jcbn.13-14 PMC3705157

[37] Owens DR Matfin G Monnier L. Basal insulin analogues in the management of diabetes mellitus: what progress have we made? Diabetes Metab Res Rev. 2014;30:104–19. 24026961 10.1002/dmrr.2469

[38] Tien KJ Hung YJ Chen JF. Basal insulin therapy: unmet medical needs in Asia and the new insulin glargine in diabetes treatment. J Diabetes Investig. 2019;10:560–70. 10.1111/jdi.12984 PMC6497775 30520564

